# Research progress on the mechanism of beta-cell apoptosis in type 2 diabetes mellitus

**DOI:** 10.3389/fendo.2022.976465

**Published:** 2022-08-18

**Authors:** SuFang You, JingYi Zheng, YuPing Chen, HuiBin Huang

**Affiliations:** ^1^ The Second Clinical Medical College of Fujian Medical University, Quanzhou, China; ^2^ Department of Endocrinology, The Second Affiliated Hospital of Fujian Medical University, Quanzhou, China

**Keywords:** type 2 diabetes, beta-cell, apoptosis, glucolipotoxicity, non-coding RNAs, exosomes, molecular mechanisms

## Abstract

Type 2 diabetes mellitus(T2DM) is regarded as one of the most severe chronic metabolic diseases worldwide, which poses a great threat to human safety and health. The main feature of T2DM is the deterioration of pancreatic beta-cell function. More and more studies have shown that the decline of pancreatic beta-cell function in T2DM can be attributable to beta-cell apoptosis, but the exact mechanisms of beta-cell apoptosis in T2DM are not yet fully clarified. Therefore, in this review, we will focus on the current status and progress of research on the mechanism of pancreatic beta-cell apoptosis in T2DM, to provide new ideas for T2DM treatment strategies.

## 1 Introduction

Diabetes mellitus (DM) is a chronic metabolic disease characterised by hyperglycemia and is prone to a variety of complications that can cause significant damage to the health of patients and place a huge economic burden on healthcare systems around the world (1). The global incidence of diabetes has increased dramatically in recent decades. International Diabetes Federation (IDF) (2) predicts that the number of people with diabetes will increase to 783.2 million globally by 2045. Type 2 diabetes (T2D) is the most prevalent form of diabetes in adults, accounting for about 95% of all people with diabetes (3). The mutual interaction between the patient’s genetic background and lifestyle and social environmental factors (e.g. obesity, physical inactivity) is closely linked to the development of T2D (4). T2D is primarily characterised by impaired insulin secretion and islet beta-cell failure (loss of beta-cell quality or function) (5). The cause of beta cell failure in T2D is still controversial. Some studies have suggested that mechanisms such as beta-cell dedifferentiation may lead to beta-cell loss (6). However, recent studies have reported that beta-cell failure is closely associated with an increase in beta-cell apoptosis (7). Butler AE et al. confirmed that (8) the mechanism of reduced beta-cell mass in patients with type 2 diabetes is increased beta-cell apoptosis through a study of 124 autopsied pancreatic tissues.

Nevertheless, the molecular mechanisms of beta-cell apoptosis are still obscure and need to be further explored. Current *in vivo* beta-cell apoptosis assays have less human evidence available due to the rapid removal of apoptotic cells and the increasing difficulty of obtaining human islets. In this paper, we will present the latest insights on the molecular mechanisms of beta-cell apoptosis in T2DM, such as glucolipotoxicity, amyloid deposition, exosome and noncoding RNAs, which would act synergistically. Moreover, we will concentrate on the effect of stress pathways induced by the above factors, including oxidative stress, endoplasmic reticulum stress, mitochondrial dysfunction, inflammation, and impaired autophagy, which may not immediately lead to beta-cell apoptosis, but whose accumulative effect over time will aggravate the deleterious effects of each pathway. In conclusion, the purpose of this paper is to provide a summary of the latest insights on the molecular mechanisms of beta-cell apoptosis in T2DM, to better master the mechanisms of beta-cell apoptosis, thus providing new ideas for identifying new therapeutic targets for T2DM.

## 2 Glucotoxicity or glucolipotoxicity and islet amyloid polypeptide

The term lipotoxicity was first introduced by Lee (9) and colleagues to describe the association of increased free fatty acids (FFA) with beta-cell function and T2D progression in the context of obesity-related T2D. Lipotoxicity has now been further defined as the detrimental effect of high concentrations of lipids and their derivatives, manifested as the excessive concentration of lipids in the cells of non-adipose tissue, resulting in impaired pancreatic beta-cell function and ultimately apoptosis (10). The harmful effects of fatty acids on human and animal beta cells have been demonstrated *in vivo* and *in vitro* (11, 12).

Free fatty acids can be categorized into unsaturated and saturated fatty acids, while different types of fatty acids have different mechanisms of action on beta-cell. Studies have revealed that long-term exposure to high levels of saturated fatty acids(e.g. palmitic acid) may lead to beta-cell malfunctions and induce apoptosis, whereas chronic exposure to unsaturated fatty acids (e.g. oleic acid) does not impair beta-cell function, and even inhibits the pro-apoptotic effects of saturated fatty acids (13). Palmitic acid (PA), also known as palmitate, is the most commonly found saturated fatty acid in plasma, it is also the primary fatty acid involved in lipotoxicity-induced beta-cell apoptosis (13). The main manifestation is that long-term exposure to PA may impair islet gene expression and beta-cell function through the triggering of the endoplasmic reticulum (ER) stress pathway and increased production of reactive oxygen species(ROS), thereby promoting beta-cell apoptosis (14). High levels of glucose have similar negative effects on beta-cell, a phenomenon known as glucotoxicity (15). However, the term “glucolipotoxicity” is now often applied, as hyperglycemia and hyperlipidemia are often present together in obesity-related T2DM, acting synergistically to accelerate the process of beta-cell apoptosis (15, 16), although controversy still exists (10).

It was found by autopsy that 90% of T2D patients had amyloid fibrin deposits in the islets, and it is often accompanied by reduced beta-cell mass (8). Therefore, islet amyloid polypeptide(IAPP)-derived amyloid deposits are characteristic of T2DM islets. IAPP is an amyloid protein that is synthesized and secreted by pancreatic beta-cell and insulin (17). Studies have shown that human IAPP (hIAPP) aggregates have toxic properties, which promote beta-cell apoptosis and islet inflammation in T2D (18). It can be confirmed in a study conducted by Tomita et al. (19)on the pancreas of type 2 diabetic patients. The mechanism of action of hIAPP is similar to glucolipotoxicity: increased expression of hIAPP disrupts autophagy and the ubiquitin-protease system (20), triggers stress pathways, thus leading to apoptosis (21).

**Figure 1:**
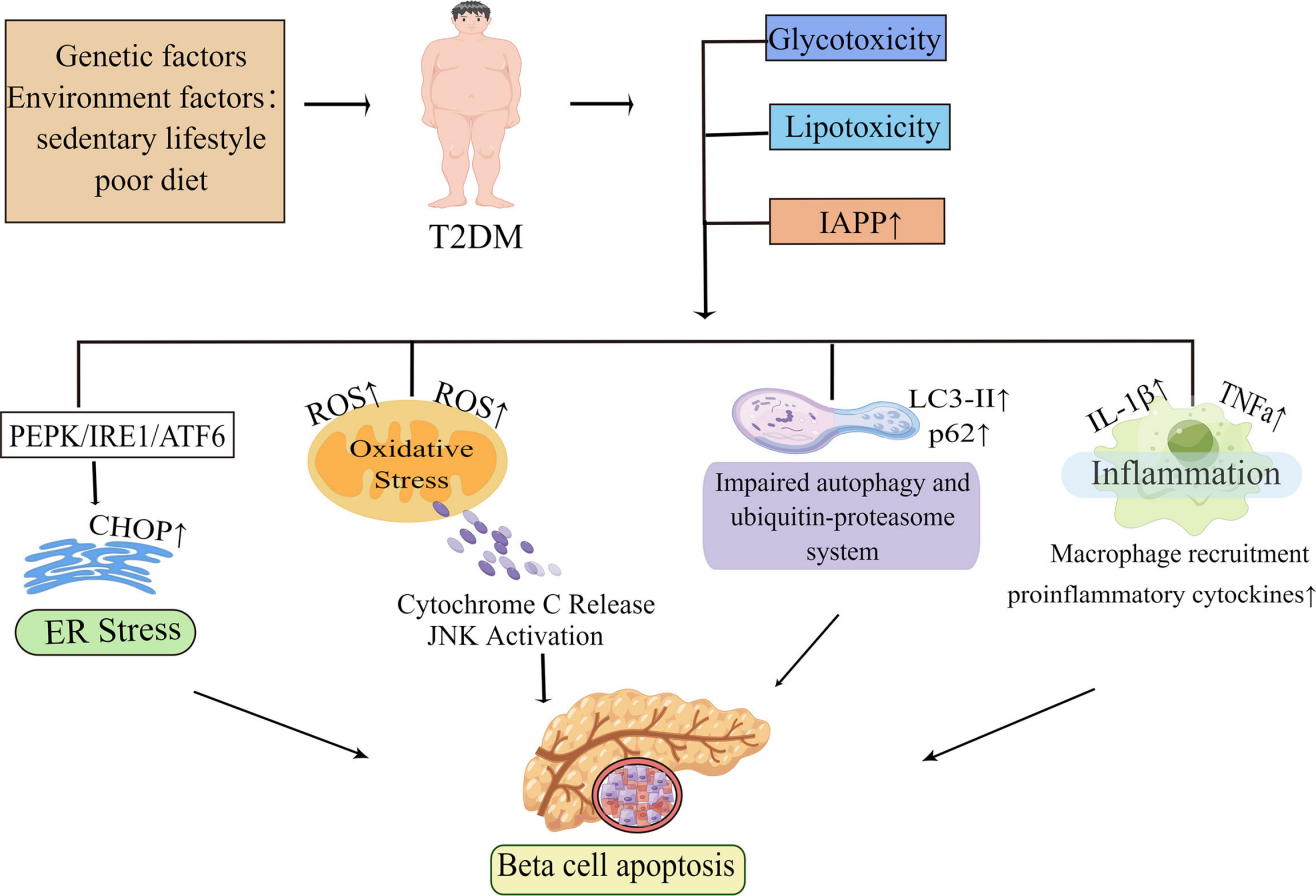
Molecular mechanisms of beta-cell apoptosis under conditions of IAPP aggregation and glucolipotoxicity. In obesity-associated type 2 diabetes, elevated islet amyloid polypeptide (IAPP), glucotoxicity, lipotoxicity and glucolipotoxicity are the most studied causative factors of beta-cell apoptosis. These factors activate all ER stress pathways in beta-cells, namely the PKR-like ER kinase (PERK), inositol-requiring enzyme 1 (IRE1), and activating transcription factor 6 (ATF6) pathways, and subsequently induce ER stress. When ER stress is prolonged or excessive, it may mediate beta-cell dysfunction and apoptosis by increasing the expression of the pro-apoptotic factor CHOP. In the presence of these pathogenic factors, it also leads to mitochondrial dysfunction and increases the production of reactive oxygen species (ROS). The increase of ROS activates the apoptotic pathway mediated by oxidative stress and mitochondrial cytochrome C in beta-cell. Autophagy can degrade damaged or misfolded cellular components and proteins under normal physiological conditions. However, under conditions of glucolipotoxicity and increased IAPP, beta-cells are subjected to sustained metabolic stress that leads to impaired autophagy which ultimately exacerbates beta-cell dysfunction thereby leading to apoptosis. Islet inflammation often occurs during T2D development and is characterized by macrophage recruitment to infiltrating immune cells, which can lead to increased production of cytokines and chemokines such as IL-1β, TNFa, and these pro-inflammatory signals can activate apoptotic mechanisms, including ER stress and oxidative stress in beta-cells. By Figdraw (www.figdraw.com).

In summary, glucolipotoxicity and islet amyloid deposition are the most investigated pathogenic elements leading to beta-cell apoptosis. These factors trigger different stress pathways, including ER stress and oxidative stress. In addition, inflammation and proteins’ primary clearance pathways are also key pathogenic factors. These pathogenic factors and the stress pathways triggered by them interact with each other to eventually induce beta-cell apoptosis (**Figure 1**). The various stress pathways triggered by these factors will be discussed in more detail in sections.

### 2.1 Endoplasmic reticulum stress and abnormal calcium release induced apoptosis

#### 2.1.1 Endoplasmic reticulum stress

The endoplasmic reticulum(ER) is an important metabolic cellular organelle that plays a vital impact in the survival and function of various cells, including responsibility for post-translational modification of proteins, proper folding, lipid synthesis, calcium ions storage and release (22). Damage to any of these processes may result in the aggregation of misfolded or unfolded proteins in the ER lumen, thus activating the unfolded protein response (UPR), also called endoplasmic reticulum (ER) stress, which is an abnormal cellular state (23). The UPR consists of three major signaling cascades activated by three endoplasmic reticulum transmembrane protein sensors: the protein kinases RNA-like ER kinase (PERK), the type I transmembrane inositol-requiring enzyme 1 (IRE1)branch, and the activating transcription factor 6 (ATF6) initiate ER stress-related downstream genes transcription by detecting unfolded proteins in the lumen of the endoplasmic reticulum, thereby degrade misfolded and unfolded proteins in the lumen (24). In addition, UPR also transfers unfolded or misfolded proteins to the cytoplasm through the ER-related degradation (ERAD) mechanism, which is subsequently eliminated by the ubiquitin-proteasome system (25). Nevertheless, when ER stress is excessive or extended, it exhausts ER calcium stores and promotes apoptosis in pancreatic beta-cell by inducing the production of ER stress-related transcription factor C/EBP homologous protein (CHOP) (26). What’s more, ER stress markers such as X-box binding protein 1 (XBP-1), immunoglobulin heavy chain binding protein (Bip), and activating transcription factor 4 (ATF4) are also triggered in the context of glucolipotoxicity (27).

XBP-1 is an important mediator of ER stress responses. Lee et al. studied (28) beta-cell from obese ob/ob or high-fat feed mice and found that XBP1 deficiency leads to impaired compensatory insulin secretion. The study also showed that XBP1 deficiency leads to an increase in beta-cell apoptosis through diminishing the antioxidant response (28). Under non-stressful situations, PEPK, IRE1a and ATF6 combine with the molecular chaperone Bip to prevent them from activating (29). When the unfolded proteins are overloaded in the ER lumen, Bip detaches from these sensors and then activates branches of the UPR to promote cell survival (30).

##### 2.1.1.1 PERK

**Figure 2:**
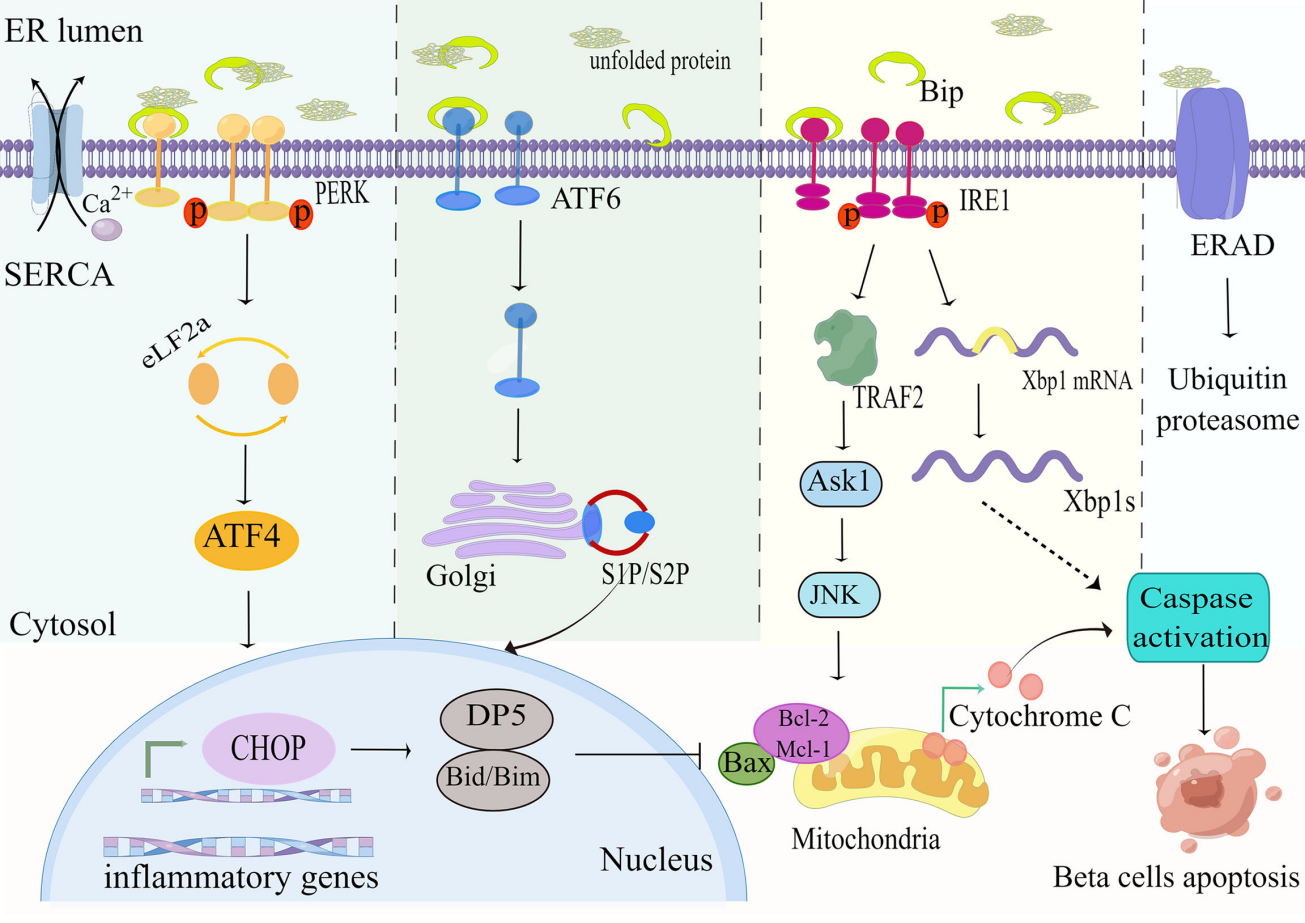
ER stress pathways. Exposure of beta-cells to palmitate leads to accumulation of unfolded or misfolded proteins and Ca^2+^depletion, which affects the folding capacity of the endoplasmic reticulum. The reduction in Ca^2+^ reserve is exacerbated by the downregulation of the sarcoendoplasmic reticulum Ca^2+^-ATPase (SERCA) pump under high glucose conditions. The misfolded protein recruits the ER chaperone BiP, leading to its separation from the ER stress sensors PERK, IRE1, and ATF6, triggering its downstream ER stress signal. This in turn upregulates the expression of the pro-apoptotic proteins CHOP, PUMA and DP5, and inhibits the anti-apoptotic members of the Bcl-2 family. These events ultimately lead to mitochondrial permeabilization, cytochrome C release, and apoptosis. By Figdraw (www.figdraw.com).

PERK is liberated from Bip and activated by diphosphorylation and autophosphorylation, which subsequently phosphorylates the downstream eukaryotic translation initiation factor 2a(eIF2a) so that it decreases the speed of protein synthesis to rectify misfolded proteins in the ER lumen (31). Furthermore, transcription factor 4 (ATF4) is activated in response to stimulation of eIF2a phosphorylation, thereby inducing the expression of pro-apoptotic C/EBP homologous protein (CHOP) to promote ER stress-mediated apoptosis (32) (**Figure 2**). It has been reported that CHOP increases the sensitivity of beta-cell to apoptosis mainly by downregulating the expression of the anti-apoptotic protein Bcl-2 (33). The Bcl-2 family is well known to include (1) anti-apoptotic proteins (e.g. BCL-2,Bcl-xL,Mcl-1); (2) pro-apoptotic proteins (e.g. Bax,Bak); and (3) pro-apoptotic BH3 members containing only the BH3 structural domain (Bad,Bid,Bim,PUMA) (34). CHOP can also induce the expression of other genes encoding apoptosis, including up-regulation of death receptor 5(DR5) (35), and tribbles-related protein3(TRB3), thereby inducing ER stress-mediated apoptosis (36).

##### 2.1.1.2 IREa

IRE1a is the most essential ER stress sensor. The IRE1a RNase domain is activated under conditions of metabolic stress (e.g. hyperglycemia, hyperlipidemia) and subsequently splices Xbp1 mRNA to generate efficient transcription factors XBP1s that upregulate the expression of ER chaperones and other UPR target genes (37) (**Figure 2**). Research reports that activation of the IRE1a-XBP1 signaling pathway degrades insulin mRNA and activates ERAD machinery to eliminate unfolded proteins from the ER (38). Interestingly, it was found that deletion of XBP1 in beta-cell of mice fed a high-fat or high-sugar diet disrupted insulin secretory capacity and increased glucolipotoxicity-mediated beta-cell apoptosis by impairing antioxidant responses (28, 39). On the other hand, it was found that activated IRE1a recruits apoptotic signal-regulating kinase 1(ASK1) by interacting with tumor necrosis factor(TNF) receptor-associated factor 2(TRAF2) to form a complex that subsequently delivers signals to c-JUN amino-terminal kinase (JNK) and p38 to promote ER stress-mediated apoptosis (40).

##### 2.1.1.3 ATF6

ATF6 is a ubiquitously expressed endoplasmic reticulum membrane-bound transcription factor. Under ER stress, the accumulation of unfolded proteins results in the resolution of BiP from ATF6, which is subsequently moved from the ER to the Golgi complex, in which it is excised by membrane-bound transcription factor site 1 and site 2 proteases (S1P and S2P) to release transcriptionally active fragments, and cleaved ATF6 acts synergistically with XBP1 to promote expression of endoplasmic reticulum chaperone genes, thereby enhancing endoplasmic reticulum folding to maintain endoplasmic reticulum homeostasis (41) (**Figure 2**).

In summary, ER stress involves multiple mechanisms. The initial aim of UPR is to promote the folding ability of the ER and reduce the load on the ER caused by unfolded proteins. However, when ER stress is excessive and the UPR is unable to cope with the load caused by the increased unfolded proteins, the JNK protein kinase and cysteine asparaginase 3,7, and 12 are activated, ultimately inducing pro-inflammatory responses and apoptosis (41, 42). Therefore, new therapeutic approaches for FFA and high glucose-induced lipotoxicity and glucotoxicity can be brought about by reducing ER stress. Glucagon-like peptide1 receptor (GLP-1R) agonists (e.g. exendin-4) were found to significantly enhance the induction of ER stress on ATF-4 and attenuate glucolipotoxicity-mediated beta-cell apoptosis in a PKA-dependent way (43). In rodent models, exendin-4 also attenuates ER stress signaling pathway by upregulating the anti-apoptotic protein JunB and the ER molecular chaperone BiP, thereby protecting beta-cell from the toxic effects of FFA (44). DB/DB and ob/ob mice of T2D treated with sodium-glucose transporter 2 inhibitors (SGLT2i) such as dapagliflozin showed reduced glycemia and increased beta-cell mass, mainly due to SGLT2i preventing glucotoxicity-induced beta-cell failure by reducing oxidative and ER stress (45).

#### 2.1.2 Abnormal calcium release

As mentioned before, the ER plays an essential factor in Ca^2+^ storage and release. Prolonged exposure to chronic palmitate or hyperglycemia, besides damaging the protein folding ability of the ER, also depletes calcium stores in the ER, thereby provoking the ER stress response (46). Ca^2+^ was observed to be released from the ER to activate the calcium-dependent pro-apoptotic protease calpain-2 in INS-1 832/13 beta-cell that had been chronically exposed to palmitate and high glucose (47). Huang et al. reported (48) that expression of h-IAPP leads to toxic oligomer formation and activates calpain-2 mediated apoptosis through increased cytosolic calcium ions. In pancreatic beta cells, maintenance of intracellular Ca^2+^ homeostasis is regulated by the activity of the ER Ca^2+^ATPases (SERCA) (49). SERCA2b overexpression has been demonstrated to prevent beta-cell from ER stress-mediated apoptosis (47). Research has reported that therapy with peroxisome proliferator-activated receptor (PPAR-γ) agonists restores islet SERCA levels and protects against beta-cell dysfunction exposed to hyperglycemic or cytokine stress conditions (50). In addition, recent studies have found that myricetin can protect beta-cell from high glucose-induced apoptosis by increasing SERCA2b expression to inhibit ER stress and Ca^2+^ efflux (51).

### 2.2 Oxidative stress and mitochondrial dysfunction-induced beta-cell apoptosis

#### 2.2.1 Oxidative stress

**Figure 3:**
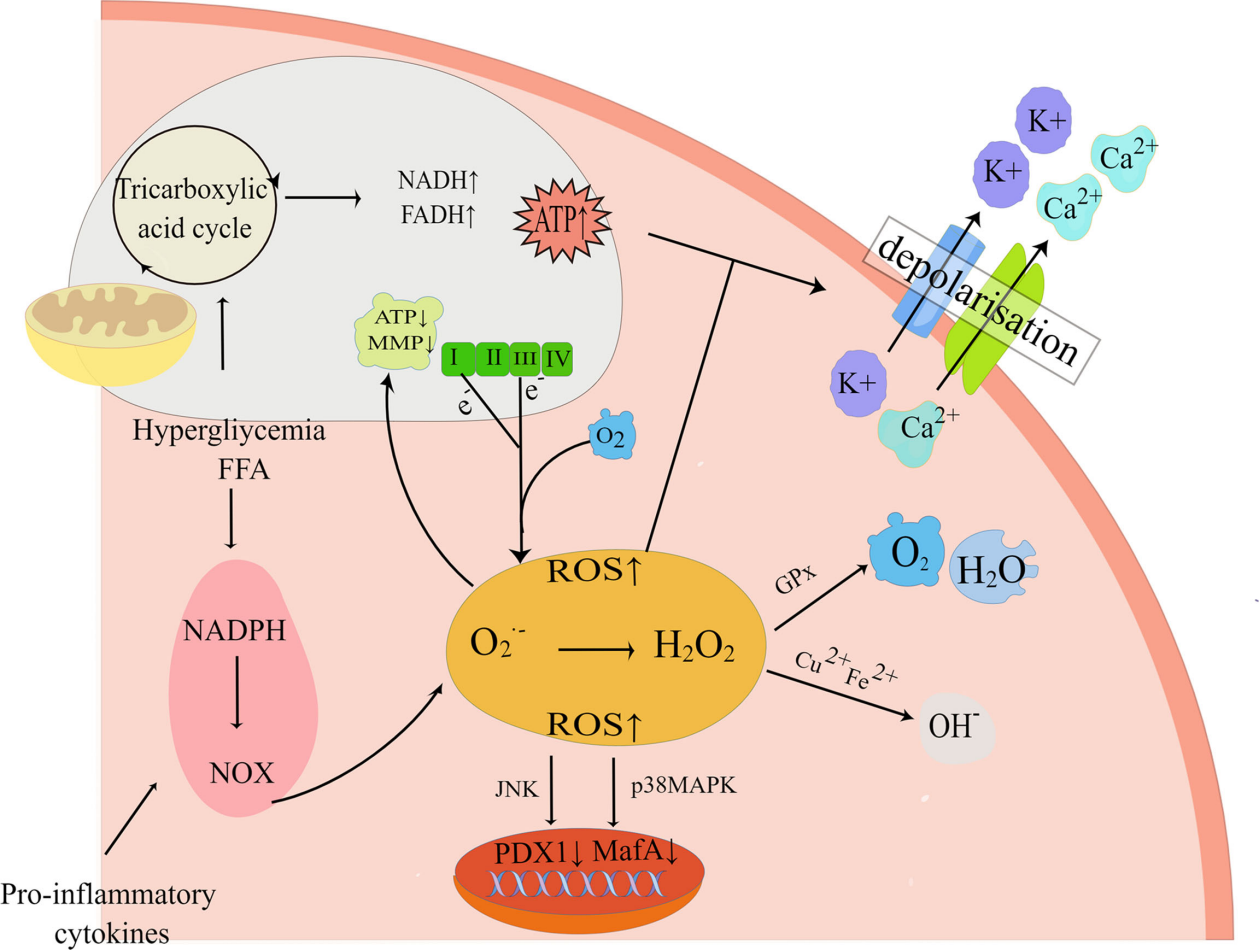
Oxidative stress pathways. Exposure to high nutrient loads including glucose (Gluc) and free fatty acids (FFA) increases the production of reactive oxygen species (ROS). If not neutralized by antioxidants, elevated ROS levels alter mitochondrial electron transfer efficiency and increase mitochondrial membrane potential (MMP), resulting in electron shedding from the normal mitochondrial electron respiratory chain transfer process. And enhanced mitochondrial metabolism increases cytoplasmic ATP synthesis, which closes ATP-sensitive K^+^ (K^+^-ATP) channels, leading to impaired insulin secretion. In addition, pro-inflammatory NADPH oxidase (NOX) activity can also elevate ROS and impair insulin gene transcription, leading to impaired insulin secretion and beta-cell apoptosis. By Figdraw (www.figdraw.com).

Substantial evidence suggests that both glucolipotoxicity and inflammation are key contributors to ROS production by beta-cell (52, 53). The potential origins of ROS include uncoupled nitric oxide synthase (NOS), the Nox family of NADPH oxidase, xanthine oxidase, and mitochondria (54). Within the physiological range, ROS and reactive nitrogen species (RNS) are known to be products of normal cellular respiration and metabolism as well as being necessary for glucose-stimulated insulin secretion (GSIS), therefore playing an influential role in insulin secretion (55). Nonetheless, in pathological conditions, excessive accumulation of ROS/RNS contributes to an imbalance in redox homeostasis, which in turn activates oxidative stress and the JNK pathway, thus taking a critical role in beta-cell apoptosis in T2DM (56). The mitochondrial electron transport chain (ETC) is usually regarded as the primary contributor to ROS in pancreatic beta-cell (57). Under glucolipotoxicity, NADH or FADH2 escapes from the ETC, resulting in electrons leakage from electron complexes I and III in the inner mitochondrial membrane, followed by the reaction of molecular oxygen with the electrons to form superoxide(O2.-), which is rapidly transformed into H2O2 (58, 59). As a matter of fact, it was shown that the accumulation of H2O2 in the ER lumen was critically important for FFA-induced ER stress, which in this way interconnects ER stress and oxidative stress (60). This interconnection would act synergistically to exacerbate the deleterious effects of glucolipotoxicity, as glucolipotoxicity-induced oxidative stress contributes to a decrease in ER Ca^2+^ and impairs ER folding capacity, while the activated ER stress can further exacerbate oxidative stress by increasing ER ROS production, which ultimately leads to beta-cell apoptosis (61). H2O2 can also diffuse intracellularly and be degraded by the antioxidant defense system of beta-cell, such as by detoxification into H2O and O2 by the antioxidant glutathione peroxidase (GPx), or elimination by peroxidase(CAT), thioredoxin(TXN) (62, 63) (**Figure 3**). Furthermore, H2O2 can be transformed into hyperactive hydroxyl radicals and OH- under the conditions of high concentrations of transition metals (e.g. Cu^2+^and Fe^2+^) (64). It has been investigated that exposure to elevated nutrient loads, comprising FFA and glucose, may lead to mitochondrial dysfunction by saturating the mitochondrial respiratory chain through the glycolytic, tricarboxylic acid cycle (TCA) pathway, which leads to greater production of NADH and FADH2, subsequently increasing the production of ROS in beta-cell (65). Consequently, oxidative stress is regarded as an essential factor in beta-cell apoptosis in T2DM. Sakuraba H et al. also confirmed (66) that the reduction in beta-cell mass was associated with oxidative stress damage through a study of islets in Japanese patients with type 2 diabetes. ROS production in beta-cell under hyperglycemia is also related to the activity of the protein kinase C (PKC) pathway (65, 67). In addition, long-term exposure to glucolipotoxicity and inflammation exacerbates oxidative stress by reducing antioxidant capacity. Alnahdi et al. (68) have shown that high levels of palmitate or glucose treatment reduced glutathione (GSH) reductase activity and increased the activity of superoxide dismutase (SOD), antioxidant enzymes, and catalase (CAT).

The harmful effects that oxidative stress has on beta-cell function also include excessive accumulation of ROS and reduced expression of islet transcription factors such as PDX1, and MafA (69). This effect is mediated by the forkhead protein transcription factor FOXO1 and the JNK pathway, while inhibition of the JNK pathway protects beta-cell from oxidative stress (70). Under oxidative stress conditions, the decrease of MafA stability is associated with the increase of GPX and p-38 mitogen-activated protein kinase (p38MAPK), so preventing p38MAPK-mediated MafA degradation can ameliorate oxidative stress-induced beta-cell apoptosis (71). Recent studies have found that glucolipotoxicity can also catalyze the formation of ROS *via* increasing the cytosolic labile iron pool(LIP), thereby triggering the onset of apoptosis (72).

#### 2.2.2 Mitochondrial dysfunction

It is known that mitochondria perform a key role in biosynthesis, Ca^2+^ homeostasis, energy metabolism, and the integration of apoptosis signals (73). What’s more, in beta-cell, nutrient sensing and insulin secretion are dependent on mitochondrial function. When beta-cell are exposed to a high nutrient environment including hyperglycemia and saturated fatty acids, mitochondrial metabolism is enhanced, and cytosolic ATP synthesis is increased, thereby closing ATP-sensitive K^+^ (K^+^-ATP) channels, resulting in depolarization of the plasma membrane, Ca^2+^ influx, and impaired insulin granule exocytosis, which further increases metabolism and oxidative phosphorylation (74). With increasing concentrations of FFA or glucose, oxidative stress produces excess ROS that impairs mitochondrial function by damaging proteins, lipids, and down-regulating subunits of the respiratory chain encoded by mitochondrial DNA(mtDNA), thus inhibiting GSIS and increasing the susceptibility of beta-cell to apoptosis (75).

Mitochondria are known to be highly flexible organelles that are in a constant state of fusion and fission (76). During this process, mitochondrial fission facilitates the isolation of dysfunctional or damaged mitochondria, which are then degraded by mitophagy (77). While PA treatment may lead to dysfunctional mitochondrial autophagy in beta-cell (78). It has been shown that palmitate-induced elevation of ROS can disrupt mitochondrial network dynamics by triggering mitochondrial breakage and inhibiting mitochondrial fusion, resulting in mitochondrial dysfunction and beta-cell apoptosis (79). Mitochondrial dysfunction contributes to increased mitochondrial ROS production, activation of nod-like receptor 3(NLRP3)-dependent pro-inflammatory responses, and thus exacerbates beta-cell apoptosis (80).

In addition to mitochondrial dysfunction, glucolipotoxicity-induced beta-cell apoptosis is also closely associated with the mitochondrial apoptotic pathway, also called the intrinsic pathway, that is mediated by Bcl-2 family proteins (75, 81). Stimulated by mitochondrial ROS and Ca^2+^ overload, mitochondrial inner membrane depolarization promotes the activation of the mitochondrial permeability transition pore(mPTP), which leads to the translocation of pro-apoptotic proteins(e.g. Bax) from the cytoplasm into the mitochondria, and simultaneously induces the release of cytochrome C from the mitochondria, and the activation of apoptotic vesicle complexes Caspase-9 and -3, inducing apoptosis (75, 82).

In summary, high glucose and palmitic acid induce oxidative stress through increased ROS and mitochondrial dysfunction, which leads to beta-cell apoptosis. What’s more, nutrient overload in glucolipotoxicity inhibits mitochondrial autophagy and ATP production. Although the specific mechanisms of oxidative stress need to be further explored, enhancing beta-cell antioxidant pathways and regulating mitochondrial function may be therapeutic targets for the protection of pancreatic beta-cell. Studies demonstrated that mitochondria-targeted antioxidants (e.g. MitoQ) can increase insulin secretion, block ROS production and reduce the performance of ER stress markers, thereby improving mitochondrial function and ER stress in pancreatic beta-cell under palmitic acid or hyperglycemia (83).

### 2.3 Autophagy pathway and ubiquitin-proteasome system induced beta-cell apoptosis

#### 2.3.1 Autophagy pathway

Autophagy, also known as macrophages, is a conserved intracellular lysosomal degradation pathway for the elimination of misfolded and or damaged proteins that are protective against various types of damage to pancreatic cells (84). It is characterized by the formation of autophagosomes (APS) around the cellular contents that are to be degraded, which then fuse with lysosomes, allowing lysosomal hydrolases to degrade the contents (85). In most cases, autophagy can prevents cell death, but when autophagy is dysregulated or deteriorates it can cause cell apoptosis. Research on animal models of T2D confirms that defective autophagic pathways lead to apoptosis of beta-cell, which is strongly related to the progression of T2DM (86). The presence of ER stress and oxidative stress is shown to trigger the autophagic pathway (87). Experiments in mice with beta-cell-specific deletion of the autophagy-related gene 7(Atg7) confirmed that autophagy is critical for the UPR and that defective autophagy in beta-cell can lead to compromised UPR and promote ER stress episodes, thereby triggering CHOP-induced beta-cell apoptosis (88). Increased autophagy markers LC3-II, Atg6, and p62 were monitored in ZDF rats, DB/DB mice, and high-fat-fed C57BL/6 mice and INS-1 cells cultured with PA and high concentrations of glucose, suggesting activation of the autophagic pathway upon prolonged exposure to high glucose or palmitate (89, 90). Another study found that treatment of pancreatic beta-cell with palmita and high concentrations of glucose inhibits the conversion of autophagy, leading to ubiquitination and accumulation of long-lived proteins, which are associated with impaired lysosomal acidification (91). Furthermore, it was observed that autophagy was activated in a JNK-dependent way and the increase in autophagosomes had a protective effect against glucolipotoxicity and h-IAPP-induced apoptosis (20, 92). However, the role of FFA on pancreatic beta autophagic flux is still controversial. Komiya (93) and colleagues reported that palmitate stimulated autophagic flux in pancreatic beta-cell *via* the JNK pathway. In contrast, Mir et al. showed (91) that glucolipotoxicity blocked autophagic flux and lead to apoptotic cell death. Consequently, the role of autophagy in type 2 diabetic beta-cell apoptosis needs further investigation.

The mammalian target of rapamycin protein (mTOR) is a critical modulator of autophagy which is activated in response to increased metabolic load (94). Studies have shown that mTOR complex 1(mTORC1) is hyperactivated and inhibits autophagic conversion under PA and high glucose treatment (94). Studies in a diabetic mouse model showed that when treated with rapamycin attenuated the negative regulation of autophagy by PA and glucose, and thus protected pancreatic beta-cell from glucolipotoxicity-induced apoptosis (95). Therefore, therapeutic interventions using drugs that target the autophagic mechanism could be used to offer new insights into the treatment of T2DM.

#### 2.3.2 The ubiquitin-proteasome system

In addition to the autophagy pathway, the ubiquitin-proteasome system(UPS) is also a major degradation pathway for keeping proteins in homeostasis. This pathway first identifies dysfunctional or misfolded proteins and then binds them covalently to ubiquitin for subsequent degradation at the proteasome. It was shown that h-IAPP disrupts the UPS, induces polyubiquitinated protein aggregation by down-regulating ubiquitin carboxy-terminal hydrolase L1(UCH-L1) activity, triggers ER stress, and elevates cleaved caspase 3 levels, thereby activating apoptosis (96). Another study reported that prolonged exposure of rat beta-cell or human islets to high levels of glucose or palmitate may trigger beta-cell apoptosis through inhibition of UPS, induction of ER stress, and dysregulation of Bcl-2 protein (97, 98).

### 2.4 Inflammation-induced apoptosis in pancreatic beta-cell

Study shows that long-term low-level inflammation is a potential mediator of T2D associated with obesity (99). This may be the result of prolonged contact with high concentrations of palmitate and glucose in human islets, characterized by accumulated immune cells and increased production of pro-inflammatory and chemotactic factors (100). Interleukin-1β (IL-1β) is a major inflammatory medium that acts through the innate immune cells to produce IL-1 type receptor (IL-1R) (101). Numerous studies have confirmed that factors like nutritional excess (high levels of glucose and FFA) and IAPP can trigger islet production of IL-1β, that IL-1β induces the pro-inflammatory factors IL-8 and IL-6, and that this pro-inflammatory response can be inhibited by the IL-1R antagonist (IL-1Ra) (102). It has been shown that FFA activates nuclear factor κB (NF-κB), JNK pathway, and activator protein-1 (Ap1) *via* Toll-like receptor 2 or 4 (TLR2/4), inducing the production of pro-inflammatory cytokines such as IL-8, IL-6 and TNF-a in beta-cells, which contribute to beta-cell apoptosis, and concurrently produces M1 phenotype macrophages, whose degradation can prevent lipotoxicity-mediated beta-cell dysfunction (103–105).. What’s more, high concentrations of glucose may also cause the production of the pro-inflammatory factor IL-1β in pancreatic beta-cells, and subsequently activates transcription factors STAT1 and NF-κB, along with upregulation of the pro-apoptotic receptor FAS to trigger beta-cell apoptosis (106). Studies using human islets and transgenic mouse islets expressing h-IAPP have indicated that IL-1β also activates caspase-8 and -3 through upregulation of the cytostatic receptor Fas, which causes apoptosis (107). By high-density microarray analysis of the beta-cell transcriptome, Bagnati et al. showed that glucolipotoxicity mediates STAT1 and NF-KB activity through tumor necrosis factor receptor 5 (TNFR5) and that selective knockdown of TNFR5 ameliorated the induction of beta-cell apoptosis by glucolipotoxicity (108). Another study revealed that PA and high glucose induce the release of mtDNA from mitochondria by decreasing mitochondrial membrane potential, and the released mtDNA leads to NLRP3 inflammatory activation, which in turn induces IL-1β production (109). In the study of pancreatic islets in DB/DB mice, the stimulator of interferon gene stimulator (STING) and interferon regulatory factor 3 (IRF3) were found to be activated, and the activated STING-IRF3 signaling pathway initiated lipotoxicity-mediated beta-cell apoptosis and inflammatory pathways (110).

Observations in rodent T2D models and human T2D patients suggest that the macrophages are the main pro-inflammatory cytokine source in pancreatic islets (111). Studies have revealed that the number of macrophages in pancreatic islets is increased, as confirmed by pancreatic sections from HFD-fed C57BL/6 mice, T2D patients, DB/DB mice, and GK rats (112). The infiltration of macrophages in the early stages may contribute to islet function, but with the progression of T2D, activated macrophages accelerate islet cell dysfunction and death (113). Macrophages have two main subpopulations: M1 and M2, the classically activated M1-like macrophages promote the generation of pro-inflammatory cytokines, including tumor necrosis factor-a(TNF-a), IL-1, and IL-6, while the alternative activated M2-like macrophages express anti-inflammatory factors, which are more involved in maintaining local homeostasis and tissue repair and remodeling (114). Mukhuty et al. found (115) that excess FFA can trigger the secretion of fetuin-A (FetA) by pancreatic beta-cells, which led to the accumulation of large numbers of islet macrophages, thereby exacerbating beta-cell dysfunction and islet inflammation. Another study found that the interaction between palmitate and hyperglycemia triggered islet secretion of S1008 calcium-binding protein A8(S100A8), which in turn promoted interactions between islet beta-cell and macrophage, thus exacerbating beta-cell apoptosis and islet inflammation (116).

In conclusion, chronic palmitate, high levels of glucose, and increased IAPP induce ER stress, which in turn activates all ER stress pathways, that is PERK, IRE1a, and ATF6 pathways. When ER stress is severe or prolonged, it can trigger beta-cell apoptosis by regulating the expression of Bcl-2 family members through mediator CHOP, and it can also enhance the formation of ROS in mitochondria and ER. And accumulated ROS can activate oxidative stress that causes endoplasmic reticulum Ca^2+^ depletion, which in turn triggers the ER stress pathway and activates the mitochondrial pathway which induces apoptosis. Furthermore, the ER stress pathway can also activate inflammatory responses and autophagic signaling by activating or inhibiting various signaling pathways, such as NF-κB signaling, p38MAPK signaling, and JNK signaling, which can also lead to pancreatic beta-cell apoptosis. In short, the various stress pathways activated by glucotoxicity, lipotoxicity or glucolipotoxicity, and IAPP can act simultaneously or synergistically, and may exacerbate the deleterious effects mediated by glucotoxicity, lipotoxicity or IAPP.

## 3 Non-coding RNAs and pancreatic beta-cell apoptosis

**Figure 4:**
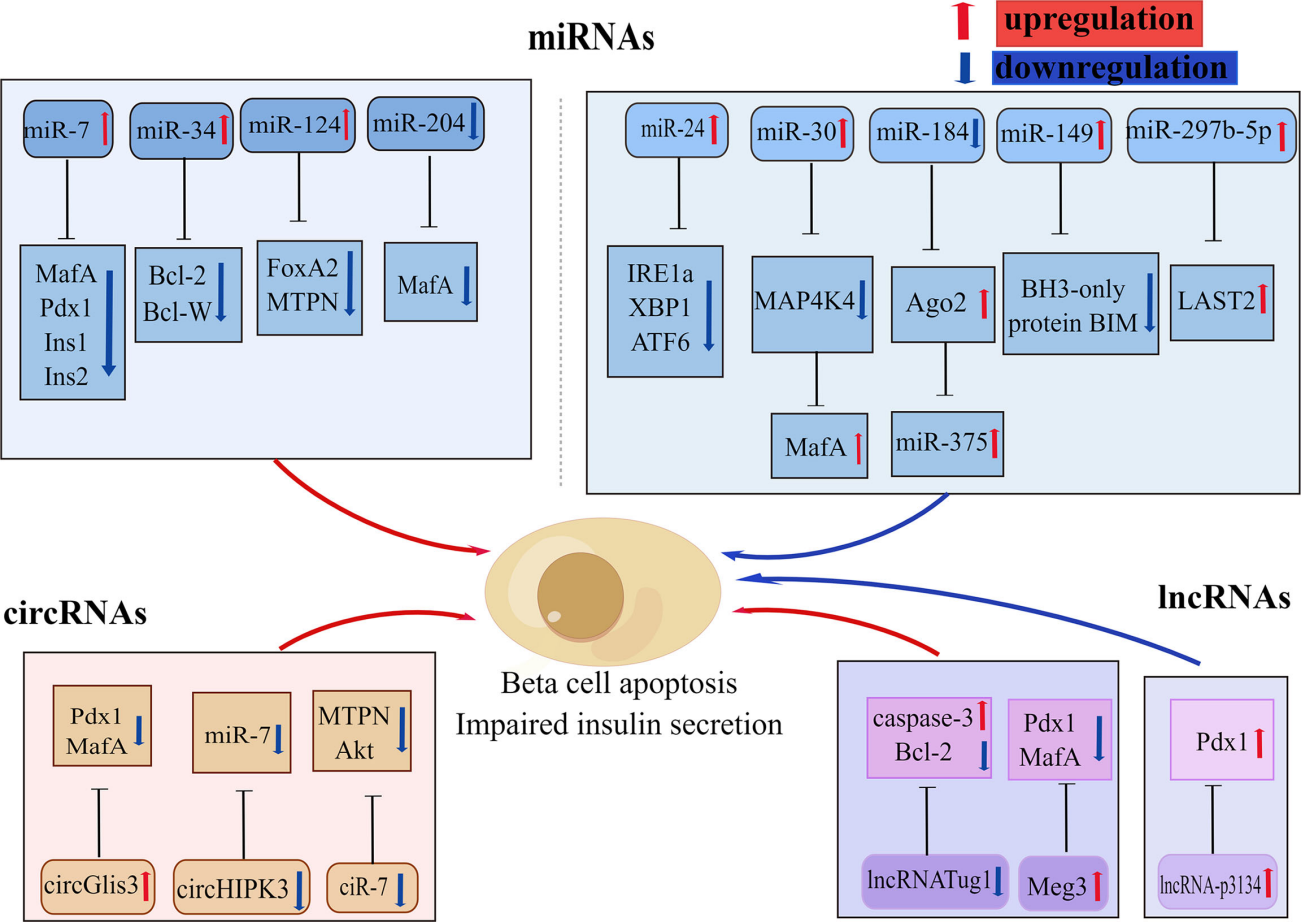
Various non-coding RNAs involved in beta-cell apoptosis. Some non-coding RNAs are involved in glucolipotoxicity and IAPP-mediated beta-cell apoptosis in type 2 diabetes by regulating the expression of beta-cell key factors. Red arrows indicate promoting, upregulation effect; blue arrows indicate inhibiting, downregulation effect. By Figdraw (www.figdraw.com).

In recent years, there has been increasing discussion on the role of non-coding RNAs (ncRNAs) in the regulation of various biological processes and metabolic diseases, particularly in obesity-induced T2D (117). NcRNAs are a type of RNA transcript that lacks coding proteins and are divided into microRNAs (miRNAs), circular RNAs (circRNAs), and long non-coding RNAs (lncRNAs). Various types of ncRNAs are involved in glucolipotoxicity -mediated beta-cell dysfunction (**Figure 4**).

### 3.1 MicroRNAs and exosomes induced beta-cell apoptosis

#### 3.1.1 MicroRNAs

In the last decade microRNA expression has become the focus of research into the genetics of T2DM susceptibility. MicroRNAs (MiRNAs) are a group of small endogenous non-coding RNAs that participate in the regulation of gene expression with specific binding to the 3’UTR region of their target mRNAs (118). A large number of mRNAs have been reported to be identified in pancreatic islets, several of which have been shown to have important effects on beta-cell function and survival, and these miRNAs change with the development of T2DM (119). In particular, miR375, miR30a, miR184, miR204, miR124, and mirR24 are all abundantly expressed in pancreatic islets (120–123). Studies have shown that miR204 can block insulin production by regulating MafA expression, leading to beta-cell dysfunction (123). Li et al. showed (124) that miR375,miR30a, and miR34a were significantly increased in rat islets and INS-1 cells that were exposed to high levels of glucose or palmitate, resulting in reduced cell survival, beta-cell dysfunction, and subsequently triggering Notch 1 pathway-mediated pancreatic beta-cell apoptosis. Another study revealed that increased expression of miR34 and miR146 in pancreatic beta-cell chronically exposed to palmitate led to insulin secretion impairment and beta-cell apoptosis, whereas suppression of miR34 and miR146 expression can block beta-cell apoptosis but could not repair normal insulin secretion (122, 125). The other important miRNA in pancreatic islets is miR7a, whose heightened expression in pancreatic islets leads to chronic hyperglycemia and compromised insulin secretion, which also promotes beta-cell failure by inhibiting beta-cell transcription factors expression (126). Recent studies have revealed that miR-297b-5p is downregulated under lipotoxicity, while upregulation of miR-297b-5p attenuates lipotoxicity-mediated beta-cell apoptosis and decreased insulin secretion through inhibiting large-tumor suppressor kinase 2 (LAST2) expression *in vitro (* 127). The expression of miR-299-5p and miR-149-5p was observed to be downregulated in rat islets, human islets, INS-1 cells, and MIN6 cells exposed to chronic sugar and palmitate, while upregulation of miR-299-5p and miR-149-5p was shown to prevent glucolipotoxicity-induced apoptosis and impaired insulin secretion (128). Among them, miR-149-5p can prevent glucotoxicity-induced beta-cell apoptosis by targeting the BH3-only protein BIM (128).

#### 3.1.2 Exosomes

Recent data suggest that miRNAs are enriched and stably expressed in exosomes (129). Virtually all kinds of human cells contain exosomes, which are extracellular vesicles (EVs) that can modulate and activate target cells through paracrine or endocrine signaling (130). Research has demonstrated that exosomes can play an essential role in cellular communication through the transfer of proteins, lipids, and ncRNA to act as messengers transmitters (131). It is interesting to note that exosomes can affect beta-cells in DM. Su et al. reported (132) that human mesenchymal stem cell-derived exosomes could alleviate type 2 diabetes by regulating peripheral insulin resistance and alleviating beta-cell destruction. Many studies have confirmed that specific enrichment of certain exosomal miRNAs is associated with beta-cell dysfunction (133). For example, Min6B1 pancreatic cells exposed to inflammatory cytokines can release miRNA-containing exosomes that are subsequently transferred to neighboring beta-cell, causing apoptosis (134). Xu et al. reported (135) that the exosome miR-26a produced by beta-cell alleviates the development of T2D by enhancing the sensitivity and beta-cell function. Screening of miRNAs in serum exosomes using high-throughput techniques after bone marrow transplantation (BMT) in mice revealed that the increased levels of miR-106b-5p and miR-222-3p were produced by myeloid cells, which were then diverted to pancreatic islet cells to induce beta-cell rejuvenation (136). In conclusion, beta-cell-derived exosomal miRNAs can be involved in T2DM development by regulating peripheral insulin sensitivity and inflammation, and are closely associated with beta-cells damage and dysfunction.

### 3.2 Circular RNAs

Circular RNAs (circRNAs) are a type of RNA that consists of a closed loop (137). The study reports that circRNAs are strongly expressed in human islets and can act as major modulators of beta-cell function and are closely related to the formation of T2D (138). They often inhibit miRNAs’ function through miRNA sponges (139). Indeed, it has been suggested that ciRS-7, which is also called CDR1, be treated as a miR-7 sponge (140). Decreased ciRS-7 levels in islets of ob/ob and DB/DB mice were observed, resulting in impaired insulin secretion and diminished beta-cell proliferation, ultimately triggering beta-cell apoptosis (140). Another miRNA sponge example is circHIPK3, which is downregulated in diabetic mouse islets and participates in beta-cell dysfunction by isolating a set of miRNAs, including miR-124-3p and miR-338-3p, and through regulating beta-cell genes (e.g.MTPN and Akt1) expression (141). A study conducted by Ren found (142) that elevated circPIP5K1A in serum samples from T2DM patients, and that circPIP5K1A competitively bound to miR-552-3p, then promoted glucolipotoxicity -induced beta-cell apoptosis *via* Janus kinase1(JAK1). In another study, circ-Tulp4 was found to be significantly downregulated in a diabetic mice model and to regulate cell growth in Min6 cells (143). In addition, this study demonstrated that overexpression of circ-Tulp4 competitively bound to mir-7222-3-p, inhibited the expression of cholesterol esterification-related gene,sterol-o-acyltransferase1(SOAT1), and activated the expression of cyclin D1, thereby enhanced beta-cell proliferation and reduced lipotoxicity-induced beta-cell apoptosis (143). Sun et al. revealed (144) that Has-circ-0054633 was abundantly expressed in diabetic and high-glucose-treated beta-cell and that inhibition of Has-circ-0054633 can ameliorate glucotoxicity-induced beta-cell apoptosis and insulin secretion *via* regulation of miR-409-3p or caspase-8. According to Cheng et al. (145) the expression of Hsa-circ-0068087 at high glucose conditions was identified to be upregulated, whereas inhibition of Hsa-circ-0068087 expression can improve the inflammation mediated by TLR4, NF-kB/NLRP3 inflammatory corpuscles under high glucose conditions through competitive binding to miR-197. In addition, circRNAs were also found to be abundant in exosomes and play a crucial role in cellular communication (146). It has been shown that the exosome circGlis3, produced by beta-cells, is markedly upregulated in conditions of glucolipotoxicity and contributes to beta-cells dysfunction through the suppression of cell survival and insulin secretion (147).

### 3.3 Long non-coding RNAs

Long non-coding RNAs (lncRNAs) are transcribed from over 200 coding nucleotides that can play primary roles in the control of gene expression through diverse molecular mechanisms (148). This is growing shreds of evidence indicating that lncRNAs are expressed in pancreatic beta-cell and play a significant role in regulating pancreatic beta-cell differentiation and proliferation, insulin synthesis and secretion, and beta-cell apoptosis. Some studies have reported that lncRNA TUG1 expression is upregulated in the pancreas and that in mice islets, downregulation of lncRNA TUG1 expression can increase beta-cell apoptosis and lead to a decrease in insulin secretion (149). Meg3 is another lncRNA that has been shown to impair GSIS by downregulating the expression of Pdx1 and MafA (150). What’s more, Meg3 also increases beta-cell apoptosis through upregulation of the pro-apoptotic proteins Bax and caspase-3 (150). Studies conducted by Ruan (151) and his colleagues found that lncRNA-p3134 expression was elevated in serum exosomes and aggregated in exosomes from diabetic patients and mouse models. This study indicates that lncRNA-p3134 actively regulates GSIS by boosting key regulators, such as Mafa in beta-cells, protects against glucotoxicity-mediated apoptosis, and maintains beta-cell mass to produce an adequate insulin secretory response (151).

In summary, miRNA, circRNA, and lncRNA are all hyper expressed in pancreatic beta-cell and can regulate critical transcription factors and other genes expression through various mechanisms, thus participating in the regulation of pancreatic beta-cell function. Nevertheless, the precise mechanism of ncRNA action in T2D remains to be elucidated.

## 4 Conclusions and perspectives

T2DM is a long-term degenerative disease that is caused by the interaction of negative lifestyles and genetic predisposition. As obesity becomes more prevalent, the incidence of T2D is growing rapidly, accounting for about 90% of all people with diabetes, which puts it as one of the most severe and provocative human health issues in the 21st century, hence the further investigation of its pathogenesis and the search for a cure have become an important goal. Numerous studies have shown beta-cell failure to be the main risk factor for the progression of T2DM, and beta-cell apoptosis is the determining factor for beta-cell failure, which suggests that if we can control the apoptosis of pancreatic beta-cells in T2DM, we can normalize the beta-cell quality of patients and fundamentally address beta-cell failure. Therefore, this review of the molecular mechanisms of beta-cell apoptosis in T2DM is presented, and the stress pathways involved are described independently (**Figure 1**). Lipotoxicity and glucotoxicity, amyloid deposition, and inflammation are all causative factors in beta-cell apoptosis and impaired insulin secretory function. Of note, the stress pathways caused by these factors, including ER stress, oxidative stress, mitochondrial dysfunction, and autophagy, are all involved in the induction of beta-cell apoptosis. These pathways may occur simultaneously at the same level, or they may interact to superimpose deleterious effects and further exacerbate beta-cell death. In addition, some non-coding RNAs also play an important role in beta-cell apoptosis with T2D patients by regulating the expression of beta-cell key factors (**Figure 4**). It is also important to notice that this review mainly summarizes the molecular mechanisms involved in beta-cell apoptosis *in vitro*, although studies on human islets are partially described, the relevance of these mechanisms remains to be confirmed in patients with T2D. In particular, it remains to be determined whether “lipotoxicity” has a significant deleterious effect on beta-cells in T2D patients.

Either exploring the mechanism of pancreatic beta-cell apoptosis occurrence at the genetic level or making progress in the research of inhibiting beta-cell apoptosis will help to reduce and slow down the occurrence and development of type 2 diabetes. For example, the traditional drug biguanides, which is the first choice for many patients with type 2 diabetes, can prevent lipotoxicity-induced beta-cell apoptosis by attenuating the ER stress response and reducing pro-apoptotic PERK/CHOP signaling (152–154). The novel therapeutic agents, GLP-1R agonists such as exendin-4 and SGLT2 inhibitors, have shown significant therapeutic efficacy in T2D treatment through enhancing beta-cell proliferation and suppressing apoptosis, but whether these agents will protect beta-cell in the clinic or the mechanism that promotes its regeneration is still unclear. Therefore, exploring the mechanisms underlying detailed beta-cell apoptosis in type 2 diabetes is important to identify new drug targets.

## Author contributions

SFY and HBH wrote the manuscript. SFY wrote the first draft of the manuscript. HHB revised the manuscript and edited. JYZ and YPC assisted in manuscript preparation. All authors contributed to the article and approved the submitted version.

## Funding

This research was supported by the Second Affiliated Hospital of Fujian Medical University horizontal scientific research project (NO: HX202201 and HX202202), the Second Affiliated Hospital of Fujian Medical University Doctoral Research Project (NO: 2021GCC03).

## Acknowledgments

The authors would like to thank Prof. HuiBin Huang for his valuable suggestions helping us to improve the manuscript. And thanks to Figdraw (www.figdraw.com) for their support in drawing the figure.

## Conflict of interest

The authors declare that the research was conducted in the absence of any commercial or financial relationships that could be construed as a potential conflict of interest.

## Publisher’s note

All claims expressed in this article are solely those of the authors and do not necessarily represent those of their affiliated organizations, or those of the publisher, the editors and the reviewers. Any product that may be evaluated in this article, or claim that may be made by its manufacturer, is not guaranteed or endorsed by the publisher.
